# Supplementary material for: Using Machine Learning Methods Combined with Vegetation Indices and Growth Indicators to Predict Seed Yield of Bromus inermis
Source: Plants (Basel). 2024 Mar 8;13(6):773. doi: 10.3390/plants13060773 (PMC10974845; doi:10.3390/plants13060773)
Supplement: Supplementary file 1 [file plants-13-00773-s001.zip › plants-2875763-supplementary.pdf]

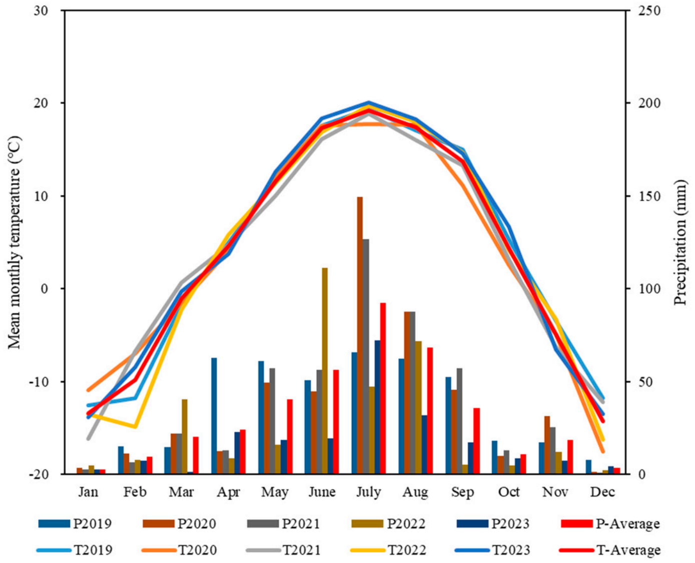

Supplementary Figure S1. Mean air temperature (℃) and monthly precipitation (mm) during 2019 to 2023.

Supplementary Table S1. The accuracy evaluation prediction results at three growth stages or the year 2023

Stage	ML	R^2	RMSE	MAE	MAPE	*P*_value
Heading Stage in 2023	RF	0.016	21.56	19.01	0.31	>0.05
Anthesis Stage in 2023	RF	0.027	109.59	84.41	0.56	>0.05
Milk Stage in 2023	RF	0.054	152.75	136.78	0.71	>0.05

Supplementary Table S2. Using RF model to Predict seed yield for the year 2023

Samples	1	2	3	4	5	6	7	8	9	10	11	12	13	14	15	16
Actual yield (kg ha^{-1})	24.1	30.2	57.1	27.3	29.7	57.7	34.8	47.7	46.6	46.5	45.3	42.9	53.0	48.2	45.9	45.9
Predicted yield (kg ha^{-1})	63.2	61.1	61.7	61.4	62.4	61.9	61.4	61.0	61.6	61.5	61.4	60.7	62.9	61.1	61.9	62.2
